# Improved cerebral oxygenation response and executive performance as a function of cardiorespiratory fitness in older women: a fNIRS study

**DOI:** 10.3389/fnagi.2014.00272

**Published:** 2014-10-08

**Authors:** Cédric T. Albinet, Kevin Mandrick, Pierre Louis Bernard, Stéphane Perrey, Hubert Blain

**Affiliations:** ^1^CeRCA (CNRS–UMR 7295), Faculty of Sport Sciences, University of PoitiersPoitiers, France; ^2^Movement to Health (M2H), Montpellier-1 University, Euromov, MontpellierFrance; ^3^University hospital of Montpellier - MacVia-LRFrance

**Keywords:** prefrontal cortex, functional near-infrared spectroscopy (fNIRS), executive functions, aerobic fitness, aging

## Abstract

Cardiorespiratory fitness has been shown to protect and enhance cognitive and brain functions, but little is known about the cortical mechanisms that underlie these changes in older adults. In this study, functional near infrared spectroscopy (fNIRS) was used to investigate variations in oxyhemoglobin [HbO_2_] and in deoxyhemoglobin [HHb] in the dorsolateral prefrontal cortex (DLPFC) during the performance of an executive control task in older women with different levels of cardiorespiratory fitness (VO_2_max). Thirty-four women aged 60–77 years were classified as high-fit and low-fit based on VO_2_max measures. They all performed a control counting (CNT) task and the Random Number Generation (RNG) task at two different paces (1 number/1 s and 1 number/1.5 s), allowing to manipulate task difficulty, while hemodynamic responses in the bilateral DLPFCs were recorded using continuous-wave NIRS. The behavioral data revealed that the high-fit women showed significantly better performance on the RNG tasks compared with the low-fit women. The high-fit women showed significant increases in [HbO_2_] responses in both left and right DLPFCs during the RNG task, while the low-fit women showed significantly less activation in the right DLPFC compared with the right DLPFC of the high-fit women and compared with their own left DLPFC. At the level of the whole sample, increases in the [HbO_2_] responses in the right DLPFC were found to mediate in part the relationship between VO_2_max level and executive performance during the RNG task at 1.5 s but not at 1 s. These results provide support for the cardiorespiratory fitness hypothesis and suggest that higher levels of aerobic fitness in older women are related to increased cerebral oxygen supply to the DLPFC, sustaining better cognitive performance.

## Introduction

Executive functions refer to a set of higher-order cognitive processes whose principal function is to facilitate behavioral adaptation to new or complex situations, specifically when routines are inappropriate. They encompass the formulation of a goal and the implementation of a strategy, planning, action sequencing and monitoring, mental flexibility, inhibition, and updating of working memory (see Anderson et al., 2008). Executive functions are critical for the activities of daily living and autonomy, but are among the most altered cognitive functions due to normal aging (West, 1996; Albinet et al., 2012). Neuroimaging and neuropsychological studies have shown that important substrates for executive functions are the frontal and parietal lobes, which are the cerebral structures that are the most vulnerable to the effects of aging (see West, 1996; Cabeza et al., 2005; Raz and Rodrigue, 2006). Cross-sectional and longitudinal studies have reported important linear age-related declines in the prefrontal volume (Raz et al., 2004, 2005) and decreased regional cerebral blood flow (rCBF) in the prefrontal cortex (PFC; Kwee and Nakada, 2003; Kalpouzos et al., 2009). These declines, however, may be modulated by lifestyle and health-related factors, such as regular physical exercise.

In the past decade, a growing body of evidence has documented the beneficial effects of physical exercise and/or cardiorespiratory fitness level on the cognitive and brain functions in older adults (for reviews see Etnier et al., 2006; Hillman et al., 2008; Voelcker-Rehage and Niemann, 2013). Aerobic exercise and cardiorespiratory fitness level, indexed by maximal oxygen uptake (VO_2_max), have been shown to be positively related to cognitive performance in older adults, particularly when executive control processes that involve the PFC are critical for task success (Kramer et al., 1999; Colcombe and Kramer, 2003; Prakash et al., 2011). The process of inhibition, a core executive function defined as the capacity to suppress irrelevant information or prepotent responses, appears particularly sensitive to the aerobic fitness level (see Smiley-Oyen et al., 2008; Colcombe et al., 2004; Boucard et al., 2012). Moreover, brain imaging studies have recently shown that greater aerobic fitness is associated with an increased volume in the frontal and parietal lobes, as well as in the hippocampus (Colcombe et al., 2003, 2006; Erickson et al., 2011), and with greater or more efficient neural activity in the prefrontal and parietal regions involved in executive functions (Colcombe et al., 2004; Prakash et al., 2011; see Voelcker-Rehage and Niemann, 2013 for a review). Indeed, using functional Magnetic Resonance Imaging (fMRI) during the performance of an executive task that involves behavioral conflict, Colcombe et al. (2004) showed that increased cardiorespiratory fitness level after a 6-month aerobic training program was associated with higher brain activations in the prefrontal and parietal cortices and significantly lower activity in the anterior cingulate cortex. Similarly, Prakash et al. (2011) reported that cardiorespiratory fitness was associated with higher activation of the prefrontal and parietal cortices, but not the posterior regions, in an inhibition task with high cognitive loads. Although the neurobiological mechanisms responsible for these benefits are not yet fully understood, converging evidence from animal and human research suggests that the release of neurotrophic factors that facilitate neurogenesis, angiogenesis, and neurovasculature may play an important role (see Cotman et al., 2007; Voss et al., 2013).

Some authors have argued that aerobic exercise and associated gains in cardiorespiratory fitness are important for cognitive and cerebral benefits to occur (see Dustman et al., 1984; Colcombe and Kramer, 2003; Colcombe et al., 2004). In this framework, the so-called *cardiorespiratory fitness hypothesis* states that increased level of aerobic fitness (high VO_2_max level) would increase rCBF (see Ainslie et al., 2008; Brown et al., 2010), thus allowing better cerebral oxygen supply to the brain, particularly in the prefrontal regions where the age-related deficit in rCBF is the most significant (Cabeza et al., 2005), and would translate into an improved executive performance. Although, as stated above, evidence suggests that increased cardiorespiratory fitness may affect brain plasticity, at a behavioral level, the direct link between cardiorespiratory fitness level and cognitive performance in older adults has not been always consistently demonstrated (see Etnier et al., 2006; Smiley-Oyen et al., 2008), deserving additional investigation.

At a neurophysiological level, the cardiorespiratory fitness hypothesis has received very little evidence. Previous studies have indirectly investigated this question by measuring rCBF or local concentration changes in paramagnetic deoxyhemoglobin [HHb] measured by the BOLD response using fMRI, but not cerebral oxygenation *per se*. Functional near infrared spectroscopy (fNIRS), which measures the hemodynamic correlates of neural activity, may help to further investigate the relationships between prefrontal oxygenation, executive performance and cardiorespiratory fitness. Functional NIRS measures changes in oxygenated and deoxygenated hemoglobin ([HbO_2_] and [HHb], respectively) from the cortical surface. The principles of fNIRS have been extensively described and appear suitable to assess the relationship between cortical activation and hemodynamic response with a high sampling rate (for reviews see Obrig and Villringer, 2003; Perrey, 2008; Ferrari and Quaresima, 2012). Recent studies have examined the advantages of fNIRS compared with fMRI in cognitive tasks (Cui et al., 2011) and have validated this technique for the study of neurovascular coupling or cognitive performance in normal aging (Vermeij et al., 2012; Fabiani et al., 2014). For example, Fabiani et al. (2014) used different brain imaging techniques during passive visual stimulation and revealed significant correlations between the [HbO_2_] response and the VO_2_max level, but not between the [HHb] or BOLD responses and VO_2_max. Moreover, these authors concluded that evaluating the functional impact of reduced neurovascular coupling in older low-fit adults requires the manipulation of the cognitive load, for example, by increasing the task difficulty in a behavioral paradigm. To the best of our knowledge, no study to date has directly tested the cardiorespiratory fitness hypothesis by measuring the changes in the blood volume and oxygenation in the brain with fNIRS during cognitive tasks in older humans. As a contribution towards a better understanding of the effects of cortical oxygenation changes in the relationship between cardiorespiratory fitness and executive performance, the present study investigated the behavioral and brain responses during cognitive tasks that challenge executive functions with different levels of difficulty in older adults.

In the present study, we evaluated the differences in executive control performance between aerobically fit older women (high VO_2_max) and aerobically unfit older women (low VO_2_max) using the Random Number Generation (RNG) task. This experimental task consists of producing random sequences of numbers at specified rates and involves executive functioning, particularly the inhibition of overlearned schemas (i.e., counting, Miyake et al., 2000; Albinet et al., 2012). Previous studies have shown that normal aging significantly impairs the behavioral performance on the RNG task (Albinet et al., 2006, 2012), while aerobic exercise or cardiorespiratory fitness improves the behavioral performance in older adults (Abou-Dest et al., 2012; Boucard et al., 2012). Different neuroimaging studies have shown that good performance on this task requires a distributed neural network in the prefrontal and parietal cortices, with a strong involvement in particular areas. The dorsolateral prefrontal cortex (DLPFC) shows a significant and systematic left-sided activation (Jahanshahi and Dirnberger, 1998; Jahanshahi et al., 1998, 2000; Joppich et al., 2004) and sometimes bilateral activation (Daniels et al., 2003; Hoshi et al., 2003; Koike et al., 2011) in normal adults. These findings oriented us to particularly examine this cortical area and to measure the changes in [HbO_2_] and [HHb] as a function of the task demands in the bilateral DLPFC (BAs 9/46). In the present study, we hypothesized that the older aerobically fit women would demonstrate better executive performance on the RNG task compared with their unfit counterparts. According to the cardiorespiratory fitness hypothesis, we expected that the older fit women would exhibit greater bilateral prefrontal activity as a function of the task being made more difficult to sustain their better executive performance compared with the unfit older women.

## Materials and methods

### Participants

Forty community-dwelling women aged 60–77 years were recruited from public meetings aimed at promoting physical activity in postmenopausal women. We focused on women because gender and hormone status have been previously shown to be potential important moderators in the relationship between fitness level and cognition and brain health (Colcombe and Kramer, 2003; Erickson et al., 2007). Thus, our sample was quite homogenous with respect to these important parameters. Women were eligible and enrolled in the study if they met the following criteria: (i) not diagnosed with any of the following conditions: rheumatoid arthritis, osteoarthritis, ischemic heart disease, severe hypertension, previous joint replacement surgery or cerebrovascular disease affecting the lower limb and cognitive functions, or a malignant tumor; (ii) no pain or medication known to alter physical and cognitive performance; and (iii) a Mini Mental State Examination (MMSE) > 26 with a memory score > 1 (Hébert et al., 1992). In order to maximize the likelihood to have an heterogeneous sample according to aerobic fitness level, all the women during the recruitment phase responded to a short validated physical activity questionnaire to indirectly estimate VO_2_max level; the 7-point Physical Activity-Rating scale from Jackson et al. (1990). Most of the low-fit women were classified as sedentary to low physically active (less than 1 h of physical activity per week). Most of the high-fit women were classified as physically active to regular exercisers (at least 150 min of moderate to vigorous physical activity per week). Before entering the study, the women were asked to provide a medical certificate ensuring there was no contraindication to performing the cardiorespiratory fitness testing. This study was approved by the local ethics Committee (CPP Sud-Méditerranée III, Number: n° 2011-A01336-35) and complied with the Declaration of Helsinki for human experimentation. All study participants provided written informed consent. Of the 40 recruited subjects, four participants did not complete the entire protocol, and two participants were discarded from the analyses due to abnormal behavioral or hemodynamics data, resulting in a total of 34 participants. All participants were right-handed according to the Edinburgh inventory (Oldfield, 1971).

### Assessment of cognitive, psychiatric and psychological status

A minimal cognitive, psychiatric and psychological assessment was assessed in all participants. The French Version of the MMSE (Hébert et al., 1992) was used to assess global cognitive functioning. The Digit Symbol-Substitution Test (DSST) from the WAIS III (Wechsler, 2000), which involves processing speed, working memory, visuomanual coordination and executive functions, was included as a descriptor of fluid intelligence. The French Version of the Mill–Hill Vocabulary Test (Part B; Deltour, 1993), which was used to assess semantic memory, is a descriptor of crystallized intelligence. Depressive symptoms were assessed using the French version of the Geriatric Depression Scale (Bourque et al., 1990), and motivation level was assessed using the French version of the Self-Motivation Inventory (André and Dishman, 2012).

### Cardiorespiratory fitness evaluation

All participants performed a maximal graded exercise test (GXT) on an electrically braked cycloergometer (Ergoselect 200P, Ergolyne, Bitz, Germany) to determine VO_2_max according to the international standards (American Thoracic Society/American College of Chest Physicians, 2003). During the exercise test, VO_2_ was measured and calculated by a breath-by-breath analysis (Ergocard^®^, MediSoft, Sorinnes, Belgium) as previously described (Gouzi et al., 2013).

### Constitution of the low-fit and the high-fit groups

The Low-fit (*n* = 17) and High-fit (*n* = 17) groups were created using the median split of the VO_2_max level. The Low-fit group had a mean VO_2_max of 20 ± 2.7 ml kg^−1^ min^−1^, which is considered a low to poor aerobic fitness level according to published norms (Shvartz and Reibold, 1990). The High-fit group had a mean VO_2_max of 29.8 ± 6.5 ml kg^−1^ min^−1^, which is considered a good to excellent aerobic fitness level according to the same published norms.

### Evaluation of executive performance

Executive performance was evaluated using the auditory RNG task developed by Albinet et al. (2006). Briefly, the participants had to say a number from one to nine aloud each time they heard a computer-generated tone, such that they generated a string of numbers as random as possible. Two paces were used to manipulate task difficulty: one tone every 1 s (fast pace) or one tone every 1.5 s (slow pace). The concept of randomness was clearly explained to the participants using the analogy of picking numbers out of a hat, and the importance of maintaining a consistent response time was emphasized. After a training period, two trials of 100 responses were recorded at each pace (thus, during 100 s for the fast pace and during 150 s for the slow pace) and then analyzed using Towse and Neil’s (1998) RgCalc software. The dependent measure, which reflects the inhibition performance for this task, was the average adjacency score from the two trials at each pace. Adjacency (A) describes the distribution of the adjacent digits (in ascending or descending series) from the ordinal sequence of alternatives (i.e., 1–2; or 8–7–6) and is expressed as a percentage score. The adjacency scores ranged between 0% (no neighboring pair) and 100% (only neighboring pairs); thus, the greater the score, the poorer the executive performance. This score was chosen because it is a reliable measure of the capacity to actively inhibit the natural strong tendency to count as a function of temporal constrains (Towse and Neil, 1998; Miyake et al., 2000), and because it is sensitive both to aging and fitness effects (Abou-Dest et al., 2012; Albinet et al., 2012; Boucard et al., 2012). As the control task, the participants were asked to repeatedly count out in order from one to nine at the same two paces and during the same period (count (CNT) task).

### fNIRS data collection and processing

A spatially resolved continuous wave spectrophotometer (NIRO-200, Hamamatsu Photonics K.K., Japan) was used to measure the time course of the relative changes in the concentrations of [HbO_2_] and [HHb]. The sampling rate was set at 6 Hz. Two pairs of optodes with an inter-optode distance of 40 mm were bilaterally placed according to the international EEG 10–20 system over the right and left DLPFCs (BAs 9/46). To reduce artifacts (motion and physiological), the participants were asked to minimize head and body movements, were given instructions to breathe gently and regularly, and were instructed to rest quietly. Pre-processing and processing of NIRS signals were performed off-line using a customized code implemented in Matlab 7.0 software (The Mathworks Inc., MA, USA). The typical hemodynamic response shows an increase for [HbO_2_] with neural activity while [HHb] signal typically behaves opposite. This hemodynamic response is often used in the feature extraction. The raw NIRS data were filtered using a first-order Butterworth low-pass filter zero-lag (cut-off frequency of 0.7 Hz) to remove the heart rate signal (Huppert et al., 2009). No detrending method was applied on the raw NIRS signals based on no important low frequency signal drift for each individual time series. From the resulting individual signals, changes (Δ) in [HbO_2_] and [HHb] were calculated for each condition with two processing methods. First, we used the slope method (see more details in Mandrick et al., 2013a) in 100-s stimulation windows as a simple, but effective feature to detect both the global hemodynamic response amplitude and the time to reach plateau level during sustained activation. The relationship between neural activity and the hemodynamic responses is often approximated to that of a linear system (Friston et al., 1994), meaning that integrated neural firing rate was assumed to be a rectangular function corresponding to the constant stimulus over time. For each experimental condition, the two trials were averaged; the dependent measures included the slope coefficients for Δ[HbO_2_] and Δ[HHb] in µM.cm/s. As suggested by Mandrick et al. (2013a) the slope coefficient of a straight line fitted to the data in a windows as feature, indicates the magnitude and the direction of the oxygenation responses over the stimulation period for each condition. Even if the issue of modeling and determining the shape of the hemodynamic responses is beyond the scope of this study, our results show that evoked fNIRS-derived cerebral hemodynamic response to sustained cognitive tasks was translated by the slope coefficient with enough sensitivity (see Mandrick et al., 2013a; present study). Second, the oxygenation response amplitude was also analyzed as it is regularly done in the field by subtracting the level obtained at the resting state (last 10 s of the rest period) from the activation period (last 10 s of the task) after reaching a plateau for each trial (Holper et al., 2009). In case of a long stimulus period (≥1 min), the [HbO_2_] signal reaches a plateau that is maintained until the cessation of the stimulus (Heekeren et al., 1997), as we observed in the present study (see Figure 3). Overall, the results were concordant between the two methods (correlated at *r* = 0.9), but the slope method appeared to be more sensitive to discriminating the fNIRS signal changes as a function of the cognitive load and the VO_2_max level. Accordingly, we only report in the present study the data concerning the slope method.

**Figure 1:**
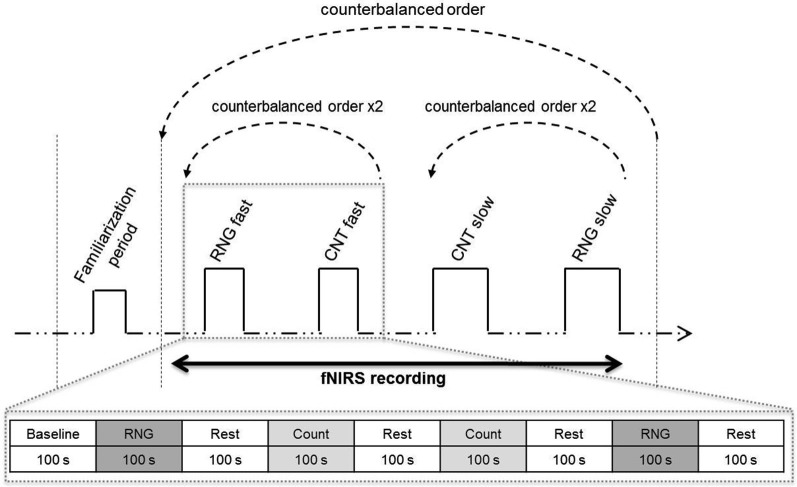
**Experimental protocol**. Upper: schema of the entire session. After the familiarization period, the participants alternated blocks of RNG tasks and count (CNT) tasks at the slow or fast pace in a counterbalanced order. [HbO_2_] and [HHb] concentrations were continuously monitored during the entire protocol using functional near-infrared spectroscopy (fNIRS). Lower: illustration of the fast pace condition. The participants performed two RNG trials alternating with rest periods and count trials in an ABBA vs. BAAB (A = RNG; B = CNT) counterbalanced order across the participants.

**Figure 2:**
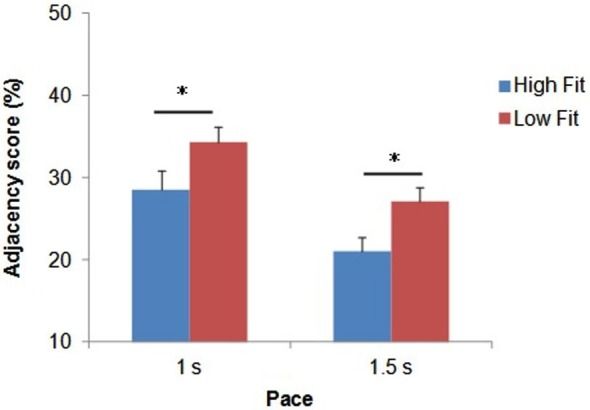
**Mean Adjacency score (in %) as a function of group and pace**. Bars represent standard-errors. *p < 0.05.

**Figure 3:**
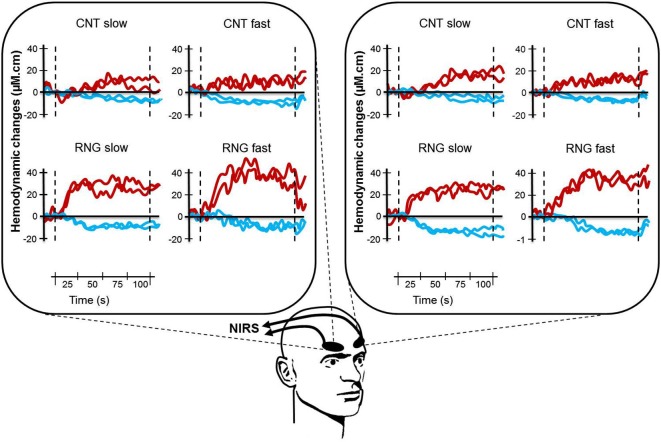
**Illustration of typical cortical activation patterns for one subject as a function of task (CNT task, upper side vs. RNG task, lower side), trial and pace (slow vs. fast) for both hemispheres (right DLPFC, left panel and left DLPFC, right panel)**. Each curve represents one trial with [HbO_2_] in red and [HHb] in blue. CNT = Count task; RNG = Random Number Generation task.

### Procedure

After careful screening for the inclusion and exclusion criteria, the participants were first evaluated for global cognitive, psychiatric and psychological status. On the second day, their cardiorespiratory fitness was measured with the GXT, which was performed at the Department of Clinical Physiology of the University Hospital. On the third assessment day, each participant was tested individually in a quiet partially obscured experimental room for the RNG performance with fNIRS recording. This session lasted approximately 50 min. After the NIRS apparatus was installed over the two DLPFCs and its functions were broadly explained, the participant was comfortably seated in an armchair. The participant performed orally the RNG task, while an experimenter visually controlled the online evolution of the hemodynamic parameters on a computer screen. After a training period to ensure familiarization with the material and the tasks, each participant performed two RNG trials at each pace alternating with rest periods and count trials in an ABBA vs. BAAB counterbalanced order across the participants (see bottom of Figure 1). The order of the paces was also counterbalanced across the participants; half of the participants started with the slow pace condition, and the other half started with the fast pace condition (see upper of Figure 1). Rest periods, which lasted the same time as the activation periods, were visually controlled for each participant to ensure that the hemodynamics variables returned to their baseline level.

### Statistical analyses

The statistical analyses were performed using STATISTICA software version 7.1 (StatSoft, France). The assumption of data normality and homogeneity was assessed using Kolmogorov-Smirnov and Levene tests, respectively. Behavioral performance on the RNG task (Adjacency score) was analyzed with a 2 (group: high-fit vs. low-fit) × 2 (pace: fast vs. slow) ANOVA with group as a between-subject factor and pace as a within-subject factor with repeated measures. For each of the hemodynamic measures, Δ[HbO_2_] and Δ[HHb], separate 2 (group: high-fit vs. low-fit) × 2 (task: RNG task vs. CNT task) × 2 (hemisphere: right vs. left) × 2 (pace: fast vs. slow) ANOVAs were performed, with group as a between-subject factor and task, hemisphere, and pace as within-subject factors with repeated measures. Mean comparisons were performed using Tukey’s HSD corrections for multiple comparisons. Finally, the relationships between executive performance, VO_2_max level and hemodynamic measures were examined using partial correlation analyses. All data are expressed as the mean ± SD. The level of significance was set at *p* < 0.05. Partial estimated effect sizes ($\eta_{p}^{2}$) were reported for significant results.

## Results

Table 1 displays the characteristics of the 34 participants of the study, that were not significantly different between the High-fit and Low-fit groups.

**Table 1:** **Characteristics of the participants**.

	High-fit group (*N* = 17)	Low-fit group (*N* = 17)	*p* value for *t*-test
**Age (years)**	67.32 (4.48)	68.88 (3.87)	*p* = 0.30
**Education (years)**	14.24 (3.68)	13.35 (2.45)	*p* = 0.42
**BMI (kg/m^2^)**	22.25 (2.51)	24.04 (2.79)	*p* = 0.06
**MMSE (max. = 30)**	29.18 (0.88)	29.12 (1.22)	*p* = 0.87
**DSST (number)**	63.41 (9.35)	61.41 (12.81)	*p* = 0.61
**Mill-Hill score (max. = 44)**	37.56 (4.62)	36.41 (3.83)	*p* = 0.44
**GDS score**	5.76 (4.47)	4.41 (3.34)	*p* = 0.32
**SMI score**	37.24 (7.12)	37.47 (4.21)	*p* = 0.91
**Systolic BP (mmHg)**	129 (17)	131 (18)	*p* = 0.73
**Diastolic BP (mmHg)**	78 (21)	75 (24)	*p* = 0.66

*Values are means (SD). Note: Education = number of years of formal education; BMI = Body Mass Index; MMSE = Mini Mental State Examination; DSST = Digit Symbol Substitution Test; GDS = Geriatric Depression Scale; SMI = Self-Motivation Inventory; BP = Blood Pressure*.

### Behavioral data

The ANOVA revealed a significant main effect of pace (*F*_(1,32)_ = 48.3; *p* < 0.0001; $\eta_{p}^{2}$ = 0.60), with higher Adjacency scores for the fast pace (31.5 ± 9.1%) compared with the slow pace (24.1 ± 7.8%). The ANOVA also revealed a significant main effect of group (*F*_(1,32)_ = 5.5; *p* < 0.05; $\eta_{p}^{2}$ = 0.15). As shown in Figure 2, whatever the pace, the high-fit group showed consistently lower Adjacency scores compared with the low-fit group, which indicated better inhibition performance.

### fNIRS data

After analyzing all NIRS signals during the RNG task, we observed that 92.3% of the activation patterns were characterized by a typical increase in Δ[HbO_2_] and a concomitant decrease in Δ[HHb]. The remaining patterns were an inverse response (0.7%) or no change (7%). During the control CNT task, we observed that 67.3% of the NIRS signals were characterized by activation patterns, 18.3% by inverse responses and 14.3% by no significant pattern detection. All those NIRS signals were included in the analyses. Figure 3 shows an illustration of typical cortical activation patterns observed in one participant as a function of trials and conditions in both right and left DLPFCs.

**Figure 4:**
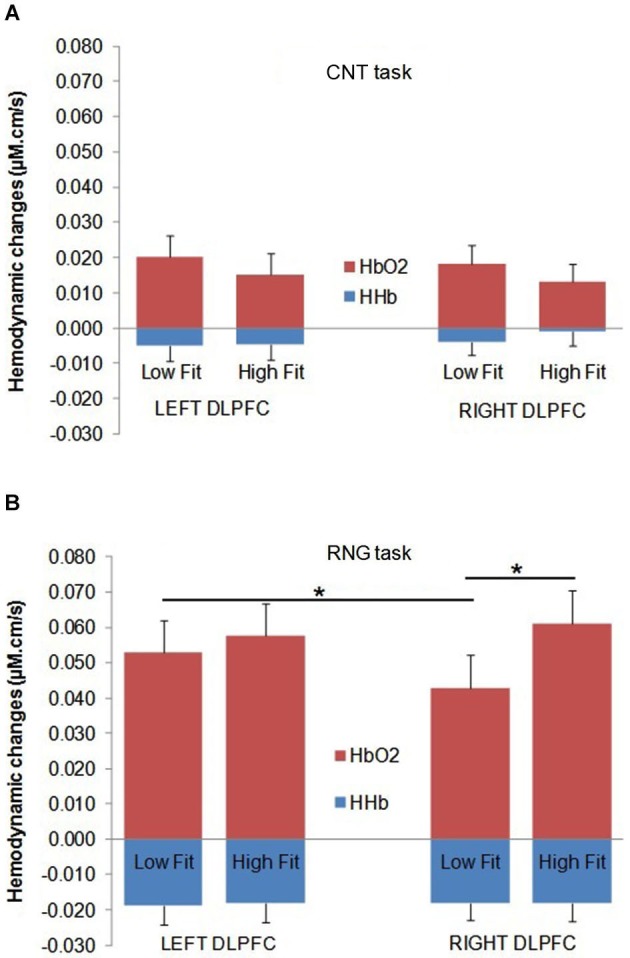
**Mean Δ[HbO_2_] slope coefficients (in red) and mean Δ[HHb] slope coefficients (in blue) in μM.cm/s during the CNT task (A) and the RNG task (B) for the Low-fit group and the High-fit group in the Left DLPFC (left panel) and in the Right DLPFC (right panel)**. Bars represent standard-errors. *p < 0.05.

The DLPFC activation during the RNG task was significantly higher compared with the control CNT task (Δ[HbO_2_]: *F*_(1,32)_ = 58.2; *p* < 0.0001; $\eta_{p}^{2}$ = 0.65; Δ[HHb]: *F*_(1,32)_ = 34.3; *p* < 0.0001; $\eta_{p}^{2}$ = 0.52. This was evidenced by a greater Δ[HbO_2_] slope coefficient for the RNG task (0.054 ± 0.037 µM.cm/s) compared with the CNT task (0.017 ± 0.022 µM.cm/s) and by a greater negative Δ[HHb] slope coefficient for the RNG task (−0.018 ± 0.020 µM.cm/s) compared with the CNT task (−0.004 ± 0.017 µM.cm/s). The DLPFC activation increased as a function of pace for the RNG task, but not for the CNT task (Δ[HbO_2_]: *F*_(1,32)_ = 4.3; *p* < 0.05; $\eta_{p}^{2}$ = 0.12; Δ[HHb]: *F*_(1,32)_ = 10.2; *p* < 0.005; $\eta_{p}^{2}$ = 0.24; see Table 2). For the CNT task, the Δ[HbO_2_] and Δ[HHb] slope coefficients were similar for both the right and left DLPFCs and for both the high-fit and low-fit groups (*p*s > 0.3; see Figure 4A). Therefore, the following results focused on the RNG task (see Table 3).

**Table 2:** **Mean (SD) Δ[HbO_2_] and Δ[HHb] slope coefficients in μM.cm/s as a function of task and pace**.

	RNG task		CNT task	
	1.5 s	1 s	1.5 s	1 s
**Δ[HbO_2_]**	0.046 (0.036)	0.061 (0.048)*	0.016 (0.023)	0.017 (0.028)
**Δ[HHb]**	−0.014 (0.021)	−0.022 (0.021)*	−0.004 (0.016)	−0.003 (0.019)

* Significantly (p < 0.05) different from 1.5 s pace.

**Table 3:** **Mean (SD) of Δ[HbO_2_] and Δ[HHb] slope coefficients in μM.cm/s as a function of task, pace, and hemisphere for the high-fit and low-fit groups**.

	High-fit group	Low-fit group
**RNG task at 1.5 s**
**Δ[HbO_2_]-Right**	0.052 (0.048)	0.031 (0.023)
**Δ[HbO_2_]-Left**	0.054 (0.042)	0.047 (0.031)
**Δ[HHb]-Right**	−0.015 (0.022)	−0.015 (0.022)
**Δ[HHb]-Left**	−0.014 (0.026)	−0.013 (0.020)
**RNG task at 1 s**
**Δ[HbO_2_]-Right**	0.070 (0.063)	0.054 (0.033)
**Δ[HbO_2_]-Left**	0.061 (0.053)	0.059 (0.041)
**Δ[HHb]-Right**	−0.022 (0.016)	−0.021 (0.025)
**Δ[HHb]-Left**	−0.023 (0.025)	−0.024 (0.024)
**CNT task at 1.5 s**
**Δ[HbO_2_]-Right**	0.013 (0.021)	0.017 (0.023)
**Δ[HbO_2_]-Left**	0.017 (0.020)	0.018 (0.031)
**Δ[HHb]-Right**	−0.001 (0.017)	−0.005 (0.018)
**Δ[HHb]-Left**	−0.007 (0.017)	−0.005 (0.017)
**CNT task at 1 s**
**Δ[HbO_2_]-Right**	0.013 (0.028)	0.020 (0.029)
**Δ[HbO_2_]-Left**	0.014 (0.029)	0.023 (0.030)
**Δ[HHb]-Right**	−0.001 (0.013)	−0.003 (0.021)
**Δ[HHb]-Left**	−0.002 (0.024)	−0.005 (0.023)

With regards to the RNG task, although there was no main effect of group or hemisphere on both Δ[HbO_2_] and Δ[HHb], the analysis yielded a significant group × hemisphere interaction on the Δ[HbO_2_] slope coefficients (*F*_(1,32)_ = 4.3; *p* < 0.05; $\eta_{p}^{2}$ = 0.12). As shown in Figure 4B, the high-fit group showed similar Δ[HbO2] slope coefficients for both the right and left DLPFCs (*p* > 0.05), whereas participants in the low-fit group showed a significantly lower Δ[HbO2] slope coefficient for the right DLPFC compared with the right DLPFC of the high-fit participants and compared with their own left DLPFC (*p* < 0.05). This interaction was not significant for Δ[HHb] (*p* > 0.8). As shown in Figure 4B, the Δ[HHb] slope coefficients were similar for both the right and left DLPFCs and for both the high-fit and low-fit groups. Finally, the analysis yielded a significant hemisphere × speed interaction (*F*_(1,32)_ = 11.3; *p* < 0.005; $\eta_{p}^{2}$ = 0.26). *Post hoc* analyses showed that the Δ[HbO_2_] slope coefficient was significantly greater in the left DLPFC (0.051 ± 0.037 µM.cm/s) compared with the right DLPFC (0.042 ± 0.037 µM.cm/s; *p* < 0.05) at the slow pace (1.5 s). At the fast pace (1 s), the Δ[HbO_2_] slope coefficients were greater than the slow pace, but they did not significantly differ between the left (0.060 ± 0.047 µM.cm/s) and the right (0.062 ± 0.050 µM.cm/s) DLPFCs (*p* > 0.05). However, this last pattern of results was similar for both the high-fit and low-fit groups, as the group × hemisphere × pace interaction was not significant (*F* < 1).

### Relationships between executive performance, VO_2_max level and cerebral oxygenation

**Figure 5:**
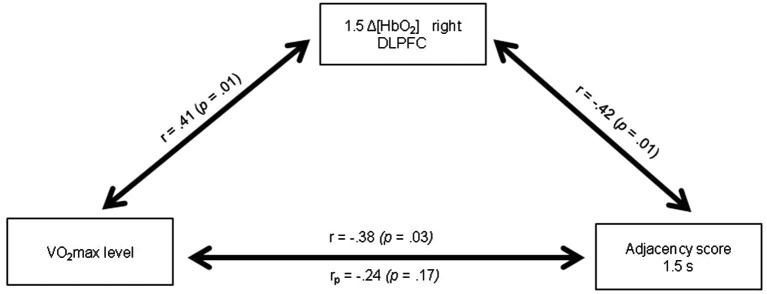
**Relationships between the VO_2_max level, the Δ[HbO_2_] slope coefficient in the right DLPFC during the 1.5 s RNG task and the Adjacency score at 1.5 s**. *r_p_*: partial correlation after controlling for the 1.5 Δ[HbO_2_] slope coefficient in the right DLPFC.

Table 4 presents the matrix of correlations between the Adjacency scores, the Δ[HbO_2_] parameters, the VO_2_max level, and potential confounders. The VO_2_max level was significantly correlated with the executive performance at the pace of 1.5 s (*r* = 0.38; *p* < 0.05) and at the pace of 1 s (*r* = 0.42; *p* < 0.05). These correlations remained significant even after controlling for age and BMI (*r*_p_ = 0.37; *p* < 0.05 and *r*_p_ =0.40; *p* < 0.05, respectively). Only VO_2_max, 1.5HbO_2_-Right and Adjacency-1.5 were significantly correlated each other, highlighting their functional significance. Figure 5 depicts the relationships between these three variables. The significant relationship between the VO_2_max level and the Adjacency score at the 1.5 s pace condition (*r* = −0.38; *p* < 0.05) no longer remained significant after controlling for Δ[HbO_2_] (*r_p_* = 0.24; *p* > 0.1). Finally, as stated above, there was a significant correlation between the VO_2_max level and the Adjacency score at 1 s, but these variables were not significantly correlated with the Δ[HbO_2_] parameters at pace 1 s in the right or left DLPFC (all *p*s > 0.4; see Table 4).

**Table 4:** **Correlation matrix between the measures of executive performance, the hemodynamic data and the characteristics of the participants**.

	Age	VO_2_max	Education	BMI	Adjacency-1.5	Adjacency-1	1.5HbO_2_	1HbO_2_	1.5HbO_2_	1HbO_2_							-Right	-Right	-Left	-Left
**Age**	–
**VO_2_max**	−0.37*	–
**Education**	−0.32	0.17	–
**BMI**	0.12	−0.59***	−0.28	–
**Adjacency-1.5**	0.02	−0.38*	−0.02	0.18	–
**Adjacency-1**	0.16	−0.42*	−0.25	0.15	0.75***	–
**1.5HbO_2_-Right**	−0.26	0.41*	−0.17	−0.10	−0.42*	−0.38*	–
**1HbO_2_-Right**	0.01	0.13	−0.28	0.12	−0.18	0.05	0.60***	–
**1.5HbO_2_-Left**	−0.19	0.19	−0.26	−0.01	−0.44*	−0.31	0.82***	0.49*	–
**1HbO_2_-Left**	0.22	−0.06	−0.35*	0.24	−0.10	0.09	0.47*	0.89***	0.51*	–

Note: BMI = Body Mass Index; Right = right dorsolateral prefrontal cortex; Left = left dorsolateral prefrontal cortex; 1.5 = RNG task at the pace of 1 number per 1.5 s; 1 = RNG task at the pace of 1 number per 1 s. *p < 0.05; **p < 0.001; ***p < 0.0001.

## Discussion

The aim of the present study was to examine whether cardiorespiratory fitness is related to better executive performance in older women and whether these cognitive benefits are related to increased prefrontal oxygenation response. In this study, we compared the functional brain activation patterns in the right and left DLPFCs in high-fit and low-fit older women during the performance of an executive controlled task, namely, the RNG task, using fNIRS. The main findings showed consistent increasing cortical activations as a function of task difficulty (i.e., the CNT vs. the RNG at a slow pace vs. the RNG at a fast pace), with significant increases in Δ[HbO_2_] and significant decreases in Δ[HHb]. Moreover, during the RNG task, the high-fit group performed better and showed greater increases in Δ[HbO_2_] bilaterally in the right and left DLPFCs, whereas the low-fit women showed significantly less activation in the right DLPFC compared with the right DLPFC of the high-fit women and compared with their own left DLPFC, which may explain, in part, their lower executive performance.

The behavioral results show that the older high-fit women demonstrated a consistently better executive performance during the RNG task at both difficulty levels (one response per 1 s and one response per 1.5 s) compared with the unfit older women. This finding confirms previous results that showed older adults who participated in regular physical activity (Abou-Dest et al., 2012) or with higher levels of aerobic fitness (Boucard et al., 2012) outperformed their sedentary or less fit counterparts on the RNG task at one response per 1 s. The present study extends these results for a less demanding condition (one response per 1.5 s) and adds to the literature on the beneficial effects of cardiorespiratory fitness on executive performance in older adults, particularly concerning inhibition (Colcombe and Kramer, 2003; Colcombe et al., 2004; Smiley-Oyen et al., 2008). Furthermore, when comparing the behavioral results of the present study with other studies using the same task, one must note that our participants’ performances were high (overall low Adjacency score); this finding suggests that our sample, regardless of the fitness level, was composed of older women with high cognitive functioning. This suggests that the positive relationship between cardiorespiratory fitness and executive functioning can be expected even in this cognitively and physically healthy population.

Consistent with previous studies, we found that the changes in [HbO_2_] were more important compared with the changes in [HHb] and that the [HbO_2_] response was more sensitive to the experimental conditions, thus most likely reflecting more direct cortical activation (Hoshi et al., 2003; Koike et al., 2011; Vermeij et al., 2012). Similar to Fabiani et al. (2014) during simple visual stimulation, we found that the VO_2_max level only correlated with the amplitude of the [HbO_2_] response, but not with the amplitude of the [HHb] response, thereby highlighting the importance of dissociating these two components of the neurovascular response. Functional NIRS results also revealed that the DLPFC activation increased with rising cognitive load for both the high-fit and low-fit participants, with minimal activation for the control CNT task (at both paces) and maximal activation for the RNG task at one response per 1 s (see Table 3). This result is in agreement with other neuroimaging studies that manipulated the cognitive load in young (Hoshi et al., 2003; Mandrick et al., 2013b) and older adults (Vermeij et al., 2012) using fNIRS.

The high-fit older women in this study showed more overall activation in the right DLPFC compared with their unfit counterparts during the RNG task. Importantly, there was no group difference in the hemodynamic response during the control CNT task, regardless of the pace, which suggests that the group difference in the hemodynamic response during the RNG task is likely not attributable to baseline differences or to difficulty in the speed elocution. Our results tend to support the hypothesis that the high-fit older women were able to recruit additional contralateral prefrontal areas to cope with task demands and that this overactivation may have contributed, in part, to their better executive performance. This explanation is in agreement with recent neuroimaging models of cognitive aging that show a possible overactivation of the prefrontal cortices and the evidence of reduced hemispheric asymmetry (HAROLD model) in some older adults (see Cabeza, 2002; Reuter-Lorenz and Lustig, 2005; Davis et al., 2008; Reuter-Lorenz and Park, 2010). According to the compensation hypothesis, this contralateral recruitment in some older adults might correspond to a form of compensatory mechanism that counteracts age-related neurocognitive deficits because it is generally associated with better cognitive performance (see Cabeza et al., 2002; Reuter-Lorenz and Cappell, 2008). Indeed, many studies have shown strongly lateralized activity in young adults and low-performing older adults, but bilateral activations in high-performing older individuals for a wide range of processes, such as executive functions (Langenecker and Nielson, 2003; Langenecker et al., 2004; Tsujii et al., 2010) or episodic memory (Cabeza et al., 2002; Gutchess et al., 2005; Angel et al., 2011). Recently, Angel et al. (2011) found that the involvement of both hemispheres in an episodic memory task increased memory performance in older adults and that older adults’ individual levels of executive functioning mediated age-related differences in the degree of lateralization of brain activity. The same mechanism may have operated in the present study for the high-fit women, who were found to respond to the cognitive load of the RNG task by recruiting the bilateral DLPFC regions. This reduced lateralization was beneficial to their executive performance, thereby supporting the compensation hypothesis. The depressed neurovascular response observed in our older low-fit women, in turn, may reflect a diminished ability of their vascular system to adapt to a task that challenges their executive functions or a reduction of their brain capillary bed (see Fabiani et al., 2014). On the whole, this pattern of results suggests that higher levels of aerobic fitness in older adults may be related to increased cerebral perfusion and better cerebral oxygen supply to the PFC (Swain et al., 2003; Ainslie et al., 2008; Brown et al., 2010). The fact that aerobic fitness was positively correlated with only the [HbO_2_] response in the present study is particularly consistent with this view.

Based on these results, we explored whether the increases in the [HbO_2_] response observed during the RNG task had a functional role in participant’s executive performance. The increase in [HbO_2_] response in both the right and left DLPFCs was positively related to the cognitive performance during the RNG task for the low pace condition (1.5 s). In contrast, the link between the cognitive performance and the DLPFC activation disappeared for the most challenging condition (RNG at 1 s; see Table 4). Taken together, these results may suggest a relationship between DLPFC activation and cognitive performance until a certain level of task difficulty and a ceiling effect for more challenging tasks. The use of a more parametric experimental design with at least three levels of difficulty would help to resolve this issue. Another possibility is that the activation of other brain regions during the most difficult condition of the RNG task may have modulated task performance. This possibility cannot be completely ruled out because the present study focused on the hemodynamic activity of the DLPFC. Nonetheless, in a PET study that used the same RNG task with six different rates (from 0.5 to 3 s per response) in six normal young adults, Jahanshahi et al. (2000) found that the left DLPFC (BAs 9, 46) was the only brain region that showed a significant increase in rCBF associated with better cognitive performance. However, in disagreement with our results, these authors found that rCBF in the DLPFC decreased with the faster rates of the RNG with a decline in cognitive performance. In our study, the increased DLPFC activation in the most difficult condition may reflect that our participants remained engaged in the task, as confirmed by their overall good performance. Clearly, these discrepancies deserve future research in this area.

One important finding of the present study was that the significant relationship between the VO_2_max level and the Adjacency score during the 1.5 s RNG task no longer remained significant after controlling for the right DLPFC [HbO_2_] changes (see Figure 5). This result indicates that the increase in the right DLPFC [HbO_2_] response may mediate, at least in part, the positive relationship between the VO_2_max level and the executive performance. Although caution is needed in the interpretation because association does not mean causality, this result provides strong support for the cardiorespiratory fitness hypothesis and has theoretical and practical implications. Indeed, it highlights the functional significance of the prefrontal increased oxygenation level in the high-fit women during a task that involves the performance of executive functions, at least for the easy RNG task condition.

The present study has some limitations that deserve further investigation. First, the study was limited to older women aged 60–77 years old, for whom executive functions are essential to prevent the decline of autonomy. As the hormone status and gender may influence the relationship between fitness and executive performance (Colcombe and Kramer, 2003; Erickson et al., 2007), our results may not be generalized to older men. Second, because we did not include a control group of younger adults, future research is warranted with a more comprehensive manipulation of the cognitive load to examine brain activation and cognitive performance as a function of age and task difficulty. In particular, it would be useful to validate in different age groups whether our results are in line with the neuroimaging models of cognitive aging we referred to in the discussion (see Cabeza, 2002; Reuter-Lorenz and Cappell, 2008). Third, the cross-sectional design of our study precludes inferences about the causality in the relationship between the VO_2_max level, the DLPFC activation and the cognitive performance. Although converging evidence from longitudinal and animal studies provides support to the model we tested, randomized-controlled trials are needed to determine whether improving aerobic fitness through exercise training may simultaneously improve prefrontal oxygenation and cognitive performance. The present study is a necessary first step, which is more cost-effective, before engaging in clinical trials. Finally, as discussed above, we only recorded cerebral hemodynamics using fNIRS in the bilateral DLPFCs, and thus, we have no information on the activation patterns in other cortical and subcortical regions involved in executive function performance. Another potential issue in the current study was the lack of a control for the skin flow contributions in our NIRS signals. Recent studies have indeed raised the question of superficial—extra-cortical—contributions in NIRS signals, specifically in the [HbO_2_] signal (Kirilina et al., 2012). In our study, the differences across the cortical areas investigated do not support the idea of a global systemic response that biased the findings. Future investigations using methods to definitively separate the cortical and extracortical signals in the NIRS signals would help to identify the precise nature of the contribution from the cortical layers in the optical signals obtained. These methods notably include the use of additional short source-detector separation optodes as regressors (Gagnon et al., 2012) and the analysis of the photon time-of-flight distribution in time-domain NIRS (Aletti et al., 2012).

In conclusion, to our knowledge, the results of the present study show for the first time that the relationship between aerobic fitness and cognitive performance for a task that involves executive functions is, at least in part, mediated by the PFC oxygenation measured by fNIRS. At both levels of task difficulty, the high-fit women showed higher patterns of [HbO_2_] response in the right DLPFC, which may explain, at least in part, their better cognitive performance; however, there was a ceiling effect concerning the relationship between the DLPFC activation and the behavioral performance. Future interventional studies are needed to examine whether physical training programs may simultaneously improve aerobic fitness, PFC oxygenation and executive function performance.

## Conflict of interest statement

The authors declare that the research was conducted in the absence of any commercial or financial relationships that could be construed as a potential conflict of interest.

## References

[B1] Abou-Dest A. Albinet C. T. Boucard G. Audiffren M. (2012). Swimming as a positive moderator of cognitive aging: a cross-sectional study with a multitask approach. J. Aging Res. 2012:273185 10.1155/2012/273185 23326664 PMC3541603

[B2] Ainslie P. N. Cotter J. D. George K. P. Lucas S. Murrell C. Shave R. (2008). Elevation in cerebral blood flow velocity with aerobic fitness throughout healthy human ageing. J. Physiol. 586, 4005–4010 10.1113/jphysiol.2008.158279 18635643 PMC2538930

[B3] Albinet C. T. Boucard G. Bouquet C. A. Audiffren M. (2012). Processing speed and executive functions in cognitive aging: how to disentangle their mutual relationship? Brain Cogn. 79, 1–11 10.1016/j.bandc.2012.02.001 22387275

[B4] Albinet C. Tomporowski P. D. Beasman K. (2006). Aging and concurrent task performance: cognitive demand and motor control. Educ. Gerontol. 32, 689–706 10.1080/03601270600835421

[B5] Aletti F. Re R. Pace V. Contini D. Molteni E. Cerutti S. (2012). Deep and surface hemodynamic signal from functional time resolved transcranial near infrared spectroscopy compared to skin flowmotion. Comput. Biol. Med. 42, 282–289 10.1016/j.compbiomed.2011.06.001 21742320

[B6] American Thoracic Society/American College of Chest Physicians (2003). ATS/ACCP statement on cardiopulmonary exercise testing. Am. J. Respir. Crit. Care Med. 167, 211–277 10.1164/rccm.167.2.211 12524257

[B7] Anderson V. Jacobs R. Anderson P. J. (2008). Executive Functions and the Frontal Lobes: A Lifespan Perspective. New York: Taylor and Francis

[B8] André N. Dishman R. K. (2012). Evidence for the construct validity of self-motivation as a correlate of exercise adherence in French older adults. J. Aging Phys. Act. 20, 231–245 22472582 10.1123/japa.20.2.231

[B9] Angel L. Fay S. Bouazzaoui B. Isingrini M. (2011). Two hemispheres for better memory in old age: role of executive functioning. J. Cogn. Neurosci. 23, 3767–3777 10.1162/jocn_a_00104 21812559

[B10] Boucard G. K. Albinet C. T. Bugaiska A. Bouquet C. A. Clarys D. Audiffren M. (2012). Impact of physical activity on executive functions in aging: a selective effect on inhibition among old adults. J. Sport Exerc. Psychol. 34, 808–827 23204360 10.1123/jsep.34.6.808

[B11] Bourque P. Blanchard L. Vézina J. (1990). Étude psychométrique de l’Échelle de dépression gériatrique. Can. J. Aging 9, 348–355 10.1017/s0714980800007467

[B12] Brown A. D. McMorris C. A. Longman R. S. Leigh R. Hill M. D. Friedenreich C. M. (2010). Effects of cardiorespiratory fitness and cerebral blood flow on cognitive outcomes in older women. Neurobiol. Aging 31, 2047–2057 10.1016/j.neurobiolaging.2008.11.002 19111937

[B13] Cabeza R. (2002). Hemispheric asymmetry reduction in older adults: the HAROLD model. Psychol. Aging 17, 85–100 10.1037//0882-7974.17.1.85 11931290

[B14] Cabeza R. Anderson N. D. Locantore J. K. McIntosh A. R. (2002). Aging gracefully: compensatory brain activity in high-performing older adults. Neuroimage 17, 1394–1402 10.1006/nimg.2002.1280 12414279

[B15] Cabeza R. Nyberg L. Park D. (2005). Cognitive Neuroscience of Aging: Linking Cognitive and Cerebral Aging. New York: Oxford University Press

[B17] Colcombe S. J. Erickson K. I. Raz N. Webb A. G. Cohen N. J. McAuley E. (2003). Aerobic fitness reduces brain tissue loss in aging humans. J. Gerontol. A Biol. Sci. Med. Sci. 58, 176–180 10.1093/gerona/58.2.m176 12586857

[B18] Colcombe S. J. Erickson K. I. Scalf P. E. Kim J. S. Prakash R. McAuley E. (2006). Aerobic exercise training increases brain volume in aging humans. J. Gerontol. A Biol. Sci. Med. Sci. 61, 1166–1170 10.1093/gerona/61.11.1166 17167157

[B19] Colcombe S. Kramer A. F. (2003). Fitness effects on the cognitive function of older adults: a meta-analytic study. Psychol. Sci. 14, 125–130 10.1111/1467-9280.t01-1-01430 12661673

[B20] Colcombe S. J. Kramer A. F. Erickson K. I. Scalf P. McAuley E. Cohen N. J. (2004). Cardiovascular fitness, cortical plasticity and aging. Proc. Natl. Acad. Sci. U S A 101, 3316–3321 10.1073/pnas.0400266101 14978288 PMC373255

[B21] Cotman C. W. Berchtold N. C. Christie L. A. (2007). Exercise builds brain health: key roles of growth factor cascades and inflammation. Trends Neurosci. 30, 464–472 10.1016/j.tins.2007.06.011 17765329

[B22] Cui X. Bray S. Bryant D. M. Glover G. H. Reiss A. L. (2011). A quantitative comparison of NIRS and fMRI across multiple cognitive tasks. Neuroimage 54, 2808–2821 10.1016/j.neuroimage.2010.10.069 21047559 PMC3021967

[B23] Daniels C. Witt K. Wolff S. Jansen O. Deuschl G. (2003). Rate dependency of the human cortical network subserving executive functions during generation of random number series-a functional magnetic resonance imaging study. Neurosci. Lett. 345, 25–28 10.1016/s0304-3940(03)00496-8 12809980

[B24] Davis S. W. Dennis N. A. Daselaar S. M. Fleck M. S. Cabeza R. (2008). Que PASA? The posterior-anterior shift in aging. Cereb. Cortex 18, 1201–1209 10.1093/cercor/bhm155 17925295 PMC2760260

[B25] Deltour J. (1993). Echelle de Vocabulaire de Mill Hill de JC Raven. Adaptation Française et Normes Européennes du Mill Hill et du Standard Progressive Matrices de Raven (PM38). Braine-le-Château: Editions l’application des techniques modernes

[B26] Dustman R. E. Ruhling R. O. Russell E. M. Shearer D. E. Bonekat H. W. Shigeoka J. W. (1984). Aerobic exercise training and improved neuropsychological function of older individuals. Neurobiol. Aging 5, 35–42 10.1016/0197-4580(84)90083-6 6738784

[B27] Erickson K. I. Colcombe S. J. Elavsky S. McAuley E. Korol D. L. Scalf P. E. (2007). Interactive effects of fitness and hormone treatment on brain health in postmenopausal women. Neurobiol. Aging 28, 179–185 10.1016/j.neurobiolaging.2005.11.016 16406152

[B28] Erickson K. I. Voss M. W. Prakash R. S. Basak C. Szabo A. Chaddock L. (2011). Exercise training increases size of hippocampus and improves memory. Proc. Natl. Acad. Sci. U S A 108, 3017–3022 10.1073/pnas.1015950108 21282661 PMC3041121

[B29] Etnier J. L. Nowell P. M. Landers D. M. Sibley B. A. (2006). A meta-regression to examine the relationship between aerobic fitness and cognitive performance. Brain Res. Rev. 52, 119–130 10.1016/j.brainresrev.2006.01.002 16490256

[B30] Fabiani M. Gordon B. A. Maclin E. L. Pearson M. A. Brumback-Peltz C. R. Low K. A. (2014). Neurovascular coupling in normal aging: a combined optical, ERP and fMRI study. Neuroimage 85(Pt. 1), 592–607 10.1016/j.neuroimage.2013.04.113 23664952 PMC3791333

[B31] Ferrari M. Quaresima V. (2012). A brief review on the history of human functional near-infrared spectroscopy (fNIRS) development and fields of application. Neuroimage 63, 921–935 10.1016/j.neuroimage.2012.03.049 22510258

[B32] Friston K. J. Holmes A. P. Worsley K. J. Poline J.-P. Frith C. D. Frackowiak R. S. J. (1994). Statistical parametric maps in functional imaging: a general linear approach. Hum. Brain Mapp. 2, 189–210 10.1002/hbm.460020402

[B33] Gagnon L. Cooper R. J. Yücel M. A. Perdue K. L. Greve D. N. Boas D. A. (2012). Short separation channel location impacts the performance of short channel regression in NIRS. Neuroimage 59, 2518–2528 10.1016/j.neuroimage.2011.08.095 21945793 PMC3254723

[B34] Gouzi F. Préfaut C. Abdellaoui A. Roudier E. de Rigal P. Molinari N. (2013). Blunted muscle angiogenic training-response in COPD patients versus sedentary controls. Eur. Respir. J. 41, 806–814 10.1183/09031936.00053512 22790908

[B35] Gutchess A. H. Welsh R. C. Hedden T. Bangert A. Minear M. Liu L. L. (2005). Aging and the neural correlates of successful picture encoding: frontal activations compensate for decreased medial-temporal activity. J. Cogn. Neurosci. 17, 84–96 10.1162/0898929052880048 15701241

[B36] Hébert R. Bravo G. Girouard D. (1992). Validation de l’adaptation française du modified mini-mental state (3MS). Rev. Geriatr. 17, 443–450

[B37] Heekeren H. R. Obrig H. Wenzel R. Eberle K. Ruben J. Villringer K. (1997). Cerebral haemoglobin oxygenation during sustained visual stimulation: a near-infrared spectroscopy study. Philos. Trans. R. Soc. Lond. B Biol. Sci. 352, 743–750 10.1098/rstb.1997.0057 9232863 PMC1691960

[B38] Hillman C. H. Erickson K. I. Kramer A. F. (2008). Be smart, exercise your heart: exercise effects on brain and cognition. Nat. Rev. Neurosci. 9, 58–65 10.1038/nrn2298 18094706

[B39] Holper L. Biallas M. Wolf M. (2009). Task complexity relates to activation of cortical motor areas during uni-and bimanual performance: a functional NIRS study. Neuroimage 46, 1105–1113 10.1016/j.neuroimage.2009.03.027 19306929

[B40] Hoshi Y. Tsou B. H. Billock V. A. Tanosaki M. Iguchi Y. Shimada M. (2003). Spatiotemporal characteristics of hemodynamic changes in the human lateral prefrontal cortex during working memory tasks. Neuroimage 20, 1493–1504 10.1016/s1053-8119(03)00412-9 14642462

[B41] Huppert T. J. Diamond S. G. Franceschini M. A. Boas D. A. (2009). HomER: a review of time-series analysis methods for near-infrared spectroscopy of the brain. Appl. Opt. 48, D280–D298 10.1364/ao.48.00d280 19340120 PMC2761652

[B42] Jackson A. S. Blair S. N. Mahar M. T. Wier L. T. Ross R. M. Stuteville J. E. (1990). Prediction of functional aerobic capacity without exercise testing. Med. Sci. Sports Exerc. 22, 863–870 10.1249/00005768-199012000-00021 2287267

[B43] Jahanshahi M. Dirnberger G. (1998). The left dorsolateral prefrontal cortex and random generation of responses: studies with transcranial magnetic stimulation. Neuropsychologia 37, 181–190 10.1016/s0028-3932(98)00092-x 10080375

[B44] Jahanshahi M. Dirnberger G. Fuller R. Frith C. D. (2000). The role of the dorsolateral prefrontal cortex in random number generation: a study with positron emission tomography. Neuroimage 12, 713–725 10.1006/nimg.2000.0647 11112403

[B45] Jahanshahi M. Profice P. Brown R. G. Ridding M. C. Dirnberger G. Rothwell J. C. (1998). The effects of transcranial magnetic stimulation over the dorsolateral prefrontal cortex on suppression of habitual counting during random number generation. Brain 121(Pt. 8), 1533–1544 10.1093/brain/121.8.1533 9712014

[B46] Joppich G. Däuper J. Dengler R. Johannes S. Rodriguez-Fornells A. Münte T. F. (2004). Brain potentials index executive functions during random number generation. Neurosci. Res. 49, 157–164 10.1016/j.neures.2004.02.003 15140558

[B47] Kalpouzos G. Chételat G. Baron J.-C. Landeau B. Mevel K. Godeau C. (2009). Voxel-based mapping of brain gray matter volume and glucose metabolism profiles in normal aging. Neurobiol. Aging 30, 112–124 10.1016/j.neurobiolaging.2007.05.019 17630048

[B48] Kirilina E. Jelzow A. Heine A. Niessing M. Wabnitz H. Brühl R. (2012). The physiological origin of task-evoked systemic artefacts in functional near infrared spectroscopy. Neuroimage 61, 70–81 10.1016/j.neuroimage.2012.02.074 22426347 PMC3348501

[B49] Koike S. Takizawa R. Nishimura Y. Marumo K. Kinou M. Kawakubo Y. (2011). Association between severe dorsolateral prefrontal dysfunction during random number generation and earlier onset in schizophrenia. Clin. Neurophysiol. 122, 1533–1540 10.1016/j.clinph.2010.12.056 21330202

[B50] Kramer A. F. Hahn S. Cohen N. J. Banich M. T. McAuley E. Harrison C. R. (1999). Ageing, fitness and neurocognitive function. Nature 400, 418–419 10.1038/22682 10440369

[B51] Kwee I. L. Nakada T. (2003). Dorsolateral prefrontal lobe activation declines significantly with age—functional NIRS study. J. Neurol. 250, 525–529 10.1007/s00415-003-1028-x 12736729

[B52] Langenecker S. A. Nielson K. A. (2003). Frontal recruitment during response inhibition in older adults replicated with fMRI. Neuroimage 20, 1384–1392 10.1016/s1053-8119(03)00372-0 14568507

[B53] Langenecker S. A. Nielson K. A. Rao S. M. (2004). fMRI of healthy older adults during Stroop interference. Neuroimage 21, 192–200 10.1016/j.neuroimage.2003.08.027 14741656

[B54] Mandrick K. Derosiere G. Dray G. Coulon D. Micallef J.-P. Perrey S. (2013a). Utilizing slope method as an alternative data analysis for functional near-infrared spectroscopy-derived cerebral hemodynamic responses. Int. J. Ind. Ergonom. 43, 335–341 10.1016/j.ergon.2013.05.003

[B55] Mandrick K. Derosiere G. Dray G. Coulon D. Micallef J. P. Perrey S. (2013b). Prefrontal cortex activity during motor tasks with additional mental load requiring attentional demand: a near-infrared spectroscopy study. Neurosci. Res. 76, 156–162 10.1016/j.neures.2013.04.006 23665138

[B56] Miyake A. Friedman N. P. Emerson M. J. Witzki A. H. Howerter A. Wager T. D. (2000). The unity and diversity of executive functions and their contributions to complex “Frontal Lobe” tasks: a latent variable analysis. Cogn. Psychol. 41, 49–100 10.1006/cogp.1999.0734 10945922

[B57] Obrig H. Villringer A. (2003). Beyond the visible—imaging the human brain with light. J. Cereb. Blood Flow Metab. 23, 1–18 10.1097/00004647-200301000-00001 12500086

[B58] Oldfield R. C. (1971). The assessment and analysis of handedness: the Edinburgh inventory. Neuropsychologia 9, 97–113 10.1016/0028-3932(71)90067-4 5146491

[B59] Perrey S. (2008). Non-invasive NIR spectroscopy of human brain function during exercise. Methods 45, 289–299 10.1016/j.ymeth.2008.04.005 18539160

[B60] Prakash R. S. Voss M. W. Erickson K. I. Lewis J. M. Chaddock L. Malkowski E. (2011). Cardiorespiratory fitness and attentional control in the aging brain. Front. Hum. Neurosci. 4:229 10.3389/fnhum.2010.00229 21267428 PMC3024830

[B61] Raz N. Gunning-Dixon F. Head D. Rodrigue K. M. Williamson A. Acker J. D. (2004). Aging, sexual dimorphism and hemispheric asymmetry of the cerebral cortex: replicability of regional differences in volume. Neurobiol. Aging 25, 377–396 10.1016/s0197-4580(03)00118-0 15123343

[B62] Raz N. Lindenberger U. Rodrigue K. M. Kennedy K. M. Head D. Williamson A. (2005). Regional brain changes in aging healthy adults: general trends, individual differences and modifiers. Cereb. Cortex 15, 1676–1689 10.1093/cercor/bhi044 15703252

[B76] Raz N. Rodrigue K. M. (2006). Differential aging of the brain: patterns, cognitive correlates and modifiers. Neurosci. Biobehav. Rev. 30, 730–748 10.1016/j.neubiorev.2006.07.001 16919333 PMC6601348

[B63] Reuter-Lorenz P. A. Cappell K. A. (2008). Neurocognitive aging and the compensation hypothesis. Curr. Dir. Psychol. Sci. 17, 177–182 10.1111/j.1467-8721.2008.00570.x

[B64] Reuter-Lorenz P. A. Lustig C. (2005). Brain aging: reorganizing discoveries about the aging mind. Curr. Opin. Neurobiol. 15, 245–251 10.1016/j.conb.2005.03.016 15831410

[B65] Reuter-Lorenz P. A. Park D. C. (2010). Human neuroscience and the aging mind: a new look at old problems. J. Gerontol. B Psychol. Sci. Soc. Sci. 65, 405–415 10.1093/geronb/gbq035 20478901 PMC2883872

[B66] Shvartz E. Reibold R. C. (1990). Aerobic fitness norms for males and females aged 6 to 75 years: a review. Aviat. Space Environ. Med. 61, 3–11 2405832

[B67] Smiley-Oyen A. L. Lowry K. A. Francois S. J. Kohut M. L. Ekkekakis P. (2008). Exercise, fitness and neurocognitive function in older adults: the “selective improvement” and “cardiovascular fitness” hypotheses. Ann. Behav. Med. 36, 280–291 10.1007/s12160-008-9064-5 18825471 PMC2748860

[B68] Swain R. A. Harris A. B. Wiener E. C. Dutka M. V. Morris H. D. Theien B. E. (2003). Prolonged exercise induces angiogenesis and increases cerebral blood volume in primary motor cortex of the rat. Neuroscience 117, 1037–1046 10.1016/s0306-4522(02)00664-4 12654355

[B69] Towse J. N. Neil D. (1998). Analyzing human random generation behavior: a review of methods used and a computer program for describing performance. Behav. Res. Methods Instrum. Comput. 30, 583–591 10.3758/bf03209475

[B70] Tsujii T. Okada M. Watanabe S. (2010). Effects of aging on hemispheric asymmetry in inferior frontal cortex activity during belief-bias syllogistic reasoning: a near-infrared spectroscopy study. Behav. Brain Res. 210, 178–183 10.1016/j.bbr.2010.02.027 20171989

[B71] Vermeij A. van Beek A. H. Olde Rikkert M. G. Claassen J. A. Kessels R. P. (2012). Effects of aging on cerebral oxygenation during working-memory performance: a functional near-infrared spectroscopy study. PLoS One 7:e46210 10.1371/journal.pone.0046210 23029437 PMC3460859

[B72] Voelcker-Rehage C. Niemann C. (2013). Structural and functional brain changes related to different types of physical activity across the life span. Neurosci. Biobehav. Rev. 37, 2268–2295 10.1016/j.neubiorev.2013.01.028 23399048

[B73] Voss M. W. Vivar C. Kramer A. F. Van Praag H. (2013). Bridging animal and human models of exercise-induced brain plasticity. Trends Cogn. Sci. 17, 525–544 10.1016/j.tics.2013.08.001 24029446 PMC4565723

[B74] Wechsler D. (2000). Manuel de L’echelle D’intelligence de Wechsler pour Adultes. Paris: Editions du Centre de Psychologie Appliquée

[B75] West R. L. (1996). An application of prefrontal cortex function theory to cognitive aging. Psychol. Bull. 120, 272–292 10.1037//0033-2909.120.2.272 8831298

